# Higher dietary phytochemical index is associated with lower odds of knee osteoarthritis

**DOI:** 10.1038/s41598-022-13019-1

**Published:** 2022-05-31

**Authors:** Farshad Amirkhizi, Seyed Mojtaba Ghoreishy, Soudabeh Hamedi-Shahraki, Somayyeh Asghari

**Affiliations:** 1 Department of Nutrition, Faculty of Public Health, Zabol University of Medical Sciences, Zabol, Iran; 2 Department of Clinical Nutrition, School of Nutritional Sciences and Dietetics, Tehran University of Medical Sciences, No#44, Hojjatdoust St., Naderi St., Keshavarz Blvd, Tehran, 141556117 Iran; 3 Department of Epidemiology and Biostatistics, Faculty of Public Health, Zabol University of Medical Sciences, Zabol, Iran

**Keywords:** Diseases, Health care, Rheumatology

## Abstract

Previous studies have shown that adherence to dietary patterns rich in plant-based foods may reduce the odds of osteoarthritis; however, limited data are available on the association of consumption of diets rich in phytochemicals and odds of knee osteoarthritis (KOA). In this case–control study conducted in Iran, we aimed to investigate whether a higher dietary phytochemical index (DPI) is associated with decreased odds of having KOA. A total of 124 cases aged 20–60 years diagnosed with bilateral primary KOA according to the American College of Rheumatology criteria and 124 controls frequency-matched on age, sex, and body mass index (BMI) were included in the study. A validated food frequency questionnaire (FFQ) was used to collect information on dietary intakes. To calculate DPI scores, the dietary energy derived from foods rich in phytochemicals (kcal) was divided by the participant’s total daily energy intake (kcal). Patients with KOA had lower intakes of dietary fiber (*P* = 0.004), vitamin A (*P* = 0.007), vitamin C (*P* = 0.001), and folate (*P* = 0.021) compared to controls. In the crude model, individuals in the third tertile of DPI had 65% lower odds of having KOA compared to those in the first tertile (OR 0.35, 95% CI 0.19 to 0.67, *P*-trend = 0.001). After adjustment for potential confounders, including age, sex, physical activity, smoking, and supplement use, this inverse association remained significant (OR 0.37, 95% CI 0.19 to 0.73, *P*-trend = 0.004). After further adjustment for BMI, this inverse association between DPI and odds of KOA also remained significant (OR 0.35, 95% CI 0.18 to 0.69, *P*-trend = 0.003). These findings suggest that adherence to a phytochemical-rich diet, as indicated by the increasing DPI score, is associated with lower odds of KOA.

## Introduction

Osteoarthritis (OA) is the most common form of arthritis characterized by joint cartilage degeneration^1,2^. OA, as a progressive disease, causes structural and functional changes in the affected joints which lead to pain and physical disability among the patients^3,4^. The prevalence of OA has been increased worldwide^1^. Approximately 20 million people in the USA are currently suffering from this disease^5^. Knee joint is one of the most important parts of the body that is affected by OA^6^. According to the current evidence, the prevalence of KOA ranges from 4% in young adults to 85% in the elderly^7^. In Iran, the prevalence in urban and rural societies is 15.3% and 19.3%, respectively^8^.

Several factors contribute to KOA, including gender, age, mechanical factors, lifestyle, and environmental factors^9,10^. The pathophysiology of KOA is affected by various factors, including diet as a modifiable component of lifestyle^11^. Weight loss in KOA has been shown to affect pain and function, but there is less information on diet quality^12^. Some observational studies have shown that higher consumption of plant-based foods and food groups including fruit and vegetables, whole grains, and dietary fiber or higher adherence to dietary patterns high in plant-based foods, might lower the odds of KOA development^13–16^. Plant-based foods contain dietary phytochemicals and it is proposed that dietary phytochemicals may be partly responsible for their protective properties^17^. Phytochemicals are natural non-nutritive bioactive compounds including polyphenols (phenolic acids, isoflavones, curcuminoids, flavonoids, terpenoids, lignans, stillbenes, and calcones), organosulfurs, and phytosterols^18^. Nuts, whole grains, legumes, and fresh fruits and vegetables contain phytochemicals^17^.

Limited studies are available linking phytochemical-rich foods to the odds of OA. A review study concluded that different types of phytochemicals might reduce the odds or progression of OA^19^.

To quantify the phytochemical content of the diet, McCarty et al. defined a simple and practical tool, known as the dietary phytochemical index (DPI). This index is defined as the percentage of daily energy intake from phytochemical-rich foods^20^. Several studies have shown the inverse association between higher DPI scores and risk of chronic diseases such as obesity, type 2 diabetes mellitus, metabolic syndrome, cancers, and cardiovascular disease^21–24^. To the best of our knowledge, no study has been performed to investigate the association between DPI and the odds of KOA. Thus, this study was aimed to assess the relationship between DPI and the odds of KOA among the Iranian population.

## Materials and methods

### Study participants

This case–control study was carried out from June 2020 to December 2020 on subjects aged 20–60 years, who were residing in Zabol County, Iran. Cases were patients referred to the hospital or private clinics with diagnosed bilateral primary KOA. Diagnosis of KOA was based on the American College of Rheumatology (ACR) clinical classification criteria for KOA including knee pain and at least one of the followings: over 50 years of age, less than 30 min of morning stiffness, crepitus on knee motion bony tenderness, bony enlargement, and no palpable warmth^25^.

Patients with a history of any rheumatic disease other than KOA, known cardiovascular diseases, diabetes mellitus, thyroid disorders, hypertension, liver diseases, kidney dysfunctions, and cancers were not included. In addition, pregnant and lactating women as well as those who were on a special diet were not included in the study. Controls were selected from subjects visiting the outpatient ophthalmology clinics of the same hospitals, who had no relationship with cases and had no history of any rheumatic disease and did not meet any of the ACR criteria for KOA. Controls were frequency-matched with cases on age (± 2 years), sex, and BMI (± 1 kg/m^2^). Subjects were not included in the control group if they had a history of rheumatoid diseases, diabetes mellitus, cardiovascular diseases, thyroid disorders, hypertension, liver diseases, kidney dysfunctions, and cancer, as well as adherence to special diets and being pregnant or lactating. Finally, 124 cases and 124 controls were eligible to participate in our study.

Data on demographic characteristics and lifestyle habits were collected using a questionnaire by trained interviewers from the cases and controls.

Before the data collection, participants were explained the aims and protocol of the research and then were asked to sign a written informed consent. The study protocol was approved by the Ethics Committee of Zabol University of Medical Sciences (Code: IR.ZBMU.REC.1398.208). The study was conducted according to the Declaration of Helsinki, and results were reported based on the strengthening the reporting of observational studies in epidemiology (STROBE) statement for case–control studies.

### Anthropometric and physical activity assessment

Anthropometric parameters were measured for all subjects. Height was measured without shoes in an upright position using a fixed non-stretchable tape with a precision of 0.1 cm. Weight was measured in light clothing by a Seca scale to the nearest 0.1 kg. BMI was calculated as weight (kg) divided by squared height (m^2^).

### Assessment of physical activity levels

Habitual physical activity (PA) levels during the past 7 days were determined based on the data obtained from the short form of the International Physical Activity Questionnaire (IPAQ)^26^. This questionnaire consists of 7 questions evaluating the frequency and duration of individuals in “vigorous”, “moderate”, and “walking” activity as well as the time spent sitting during the last week. The validity and reliability of the Persian translation of this questionnaire have been approved in Iranian populations (Cronbach’s alpha = 0.7 and test–retest reliability coefficient = 0.9) previously^26^. PA data obtained from the questionnaire were transformed into energy expenditure estimates as metabolic equivalents (METs) using published values^27^. One MET is equal to the energy expenditure of an individual during rest for 1 min and is approximately equal to 3.5 ml O_2_/kg/min in adults^27^. To convert the IPAQ data to the weekly physical activity (MET-h/week), the number of hours spent in each category was multiplied by the specific MET score for that activity^28^.

### Dietary intake assessment

In this study, expert interviewers administered a block-format-validated 123-item semi-quantitative food frequency questionnaire (FFQ) to examine dietary intakes of participants over the past year^29,30^. This questionnaire is validated among the Iranian population. Each participant reported his/her average intake of different food items (per day, week, or month) in a face-to-face interview. Considering the U.S. Department of Agriculture’s food composition database (modified for Iranian foods)^31^, daily nutrients and energy intakes were estimated using Nutritionist IV software (First Databank, Hearst Corp., SanBruno, CA, USA). A validation study^32^ revealed reasonable estimates of long-term dietary intakes for this questionnaire because good correlations were seen between dietary intakes obtained from this questionnaire and those from the average of 24-h dietary recalls (two recalls in each month of a year) as the gold standard.

### Calculation of the dietary phytochemical index

We estimated DPI using McCarty equation^33^ as the following:

$$ {\text{DPI}} = \, \left[ {{\text{Dietary energy derived from phytochemical}} - {\text{rich foods }}\left( {{\text{kcal}}} \right)/{\text{total daily energy intake }}\left( {{\text{kcal}}} \right)} \right] \times {1}00. $$

The phytochemical-rich foods which were considered in the current study are as follows: whole grains (Sangak and Barbari bread, which are traditional Iranian bread); fruits (red, yellow, and orange fruits); vegetables (dark green vegetables, red, orange vegetables, starchy vegetables, and other vegetables); soy products (soybean); nuts (peanut, almond, walnut, pistachio, and hazelnut); legumes (lentil, beans, chickpea); olives; olive oil; natural fruit and vegetable juices (carrot juice, orange juice, Limon juice). Potato, as a food item in the vegetable group, was not considered in DPI calculation due to its low content of phytochemicals^34^.

### Statistical analysis

The results were presented as mean ± standard deviation (SD) for continuous data and frequency (percent) for categorical data. To investigate the association between DPI and odds of KOA, participants were categorized based on cut-offs points of DPI score as follows: the first tertile, < 26.80; second tertile, 26.80 to 33.15; third tertile, > 33.15. The normality of the data distribution was checked using a Q–Q plot and Kolmogorov–Smirnov test. Comparison of demographic characteristics and dietary intakes between cases and controls were performed by applying independent samples t-test for continuous data and chi-square test for categorical data. Differences in the characteristics of participants across tertiles of DPI were explored by one-way analysis of variance (ANOVA) or Mantel–Hanszel extension test, as appropriate. We applied analysis of covariance (ANCOVA) to compare sex-, age-, and total energy-adjusted dietary intakes of participants across categories of DPI score. To explore the association of DPI score with KOA, binary logistic regression was applied in crude and multivariable-adjusted models. In first model, age (continuous), sex (male/female), physical activity (continuous), smoking status (yes/no), and supplement use (yes/no) were adjusted. In the second model, BMI was additionally adjusted. All statistical analyses were performed using SPSS software (version 18; SPSS, Chicago, IL, USA). *P*-values less than 0.05 were considered significant.

### Ethics approval and consent to participate

Before collecting the data, the objectives and protocol of the research were explained to the participants. The study protocol was approved by the Ethics Committee of Zabol University of Medical Sciences (Code: IR.ZBMU.REC.1398.208).

## Results

### Characteristics of participants

In this study, 248 subjects (124 cases and 124 controls) were included. The mean age and BMI of the participants were 48.7 ± 7.9 years and 28.3 ± 3.1 kg/m^2^, respectively. Totally, 45.6% (n = 113) of study participants were females and 54.4% (n = 135) were males. The DPI score of the participant’s diet ranged from 12.2 to 62.1 with a mean ± SD of 30.2 ± 7.6. Characteristics of patients with KOA (cases), controls, and all the participants across tertiles of DPI are shown in Table 1. The mean age of patients with KOA and controls was 49.2 ± 8.1 and 48.1 ± 7.6 years, respectively. No significant differences in mean age, BMI, and WC were observed between patients with KOA and controls. In addition, the distribution of participants in terms of physical activity level, educational status, smoking status, and obesity was not significantly different between cases and controls, while there was a significant difference between the two groups regarding supplement use (*P* < 0.001) and DPI score (*P* < 0.001).

**Table 1:** Characteristics of patients with knee osteoarthritis, controls and all the participants across tertiles of dietary phytochemical index (DPI).

Variables	Knee osteoarthritis		*P*^‡^	Tertiles of DPI^†^			*P*-trend^§^
	Yes (n = 124)	No (n = 124)		Tertile 1 (n = 83)	Tertile 2 (n = 83)	Tertile 3 (n = 82)	
Male, *n* (%)	58 (46.5)	55 (44.4)	0.702	39 (47.0)	39 (47.0)	35 (42.7)	0.815
Age (years)	49.2 ± 8.1	48.1 ± 7.6	0.247	49.7 ± 9.2	47.6 ± 7.4	48.7 ± 6.8	0.242
Weight (kg)	84.3 ± 13.9	82.9 ± 12.7	0.439	82.7 ± 12.4	83.6 ± 13.9	84.5 ± 13.6	0.679
BMI (kg/m^2^)	28.6 ± 3.3	27.9 ± 3.0	0.106	27.8 ± 3.1	28.4 ± 3.1	28.5 ± 3.1	0.318
WC (cm)	95.3 ± 12.8	94.9 ± 11.7	0.839	92.7 ± 12.3	95.7 ± 12.6	96.9 ± 11.7	0.080
General obesity, *n* (%)	48 (38.7)	44 (35.5)	0.599	26 (31.3)	31 (37.3)	35 (42.7)	0.319
Abdominal obesity, *n* (%)	57 (46.0)	54 (43.5)	0.702	30 (36.1)	41 (49.4)	40 (48.8)	0.153
Physical activity (MET-h/week)	36.2 ± 5.6	37.3 ± 4.9	0.088	35.9 ± 5.6	36.7 ± 5.4	37.6 ± 4.8	0.102
**Education, *n* (%)**							
Primary and no schooling	70 (56.5)	69 (55.6)	0.439	46 (55.4)	50 (60.2)	43 (52.4)	0.531
Secondary and high school	32 (25.8)	39 (31.5)		26 (31.3)	23 (27.7)	22 (26.8)	
Diploma and university	22 (17.7)	16 (12.9)		11 (13.3)	10 (12.0)	17 (20.7)	
Smokers, *n* (%)	27 (21.8)	23 (18.5)	0.527	16 (19.3)	20 (24.1)	14 (17.1)	0.516
Supplement use, *n* (%)	61 (49.2)	24 (19.4)	< 0.001	33 (39.8)	27 (32.5)	25 (30.5)	0.418
DPI	28.5 ± 7.2	31.9 ± 7.5	< 0.001	22.2 ± 3.4	30.2 ± 2.0	38.3 ± 5.1	< 0.001

Data are expressed as mean ± standard deviation unless indicated otherwise.

Min and Max of DPI in the participants were 12.2 and 62.1, respectively.

*DPI* dietary phytochemical index, *BMI* body mass index, *WC* waist circumference.

^†^Tertile cut-points of DPI are as follows: first tertile, < 26.80; second tertile, 26.80 to 33.15; third tertile, > 33.15.

^‡^Obtained from independent samples *t*-test or χ^2^ test for continuous or categorical variables, respectively.

^§^Obtained from ANOVA or Mantel-Hanszel extension test for continuous or categorical variables, respectively.

The mean (SD) of the DPI scores in the participants was 22.2 (3.4), 30.2 (2.0), and 38.3 (5.1) in the first, second, and third tertile, respectively. Comparing participants across quartiles of DPI score, we failed to find any significant difference in mean age, BMI, and WC as well as the distribution of participants in terms of sex, general and abdominal obesity, smoking status, education, and physical activity level (Table 1).

### Dietary intakes

Dietary intakes of patients with KOA, controls, and all the participants across tertiles of DPI are shown in Table 2. Patients with KOA reported lower intakes of dietary fiber (*P* = 0.004), vitamin A (*P* = 0.007), vitamin C (*P* = 0.001), and folate (*P* = 0.021) compared to controls. There were no significant differences in total energy, carbohydrate, protein, total fat, saturated fatty acids (SFA), monounsaturated fatty acids (MUFA), polyunsaturated fatty acids (PUFA), vitamin E, calcium, and zinc intakes between cases and controls. In addition, patients with KOA had lower intakes of fruits, vegetables, and olive sources than controls (all P < 0.001), whereas there were no significant differences in consumption of meats, grains, nuts, legumes, and dairy products.

**Table 2:** Dietary intakes of patients with knee osteoarthritis, controls and all the participants across tertiles of dietary phytochemical index (DPI).

Variables	Knee osteoarthritis		*P*^‡^	Tertiles of DPI^†^			*P*^§^
	Yes (n = 124)	No (n = 124)		Tertile 1 (n = 82)	Tertile 2 (n = 84)	Tertile 3 (n = 82)	
Energy intake (kcal)^#^	2275 ± 92	2218 ± 87	0.231	2116 ± 68	2292 ± 87	2487 ± 93	< 0.001
**Nutrients**							
Carbohydrate (g/day)	313 ± 23	310 ± 19	0.086	285 ± 16	312 ± 21	346 ± 27	0.001
Protein (g/day)	71.9 ± 12.8	72.3 ± 13.7	0.502	68.5 ± 12.3	74.7 ± 11.8	71.6 ± 13.7	0.182
Total fat (g/day)	94.6 ± 17.8	93.8 ± 19.3	0.215	97.5 ± 16.8	87.6 ± 15.7	96.3 ± 19.3	0.085
SFA (g/day)	31.5 ± 10.1	31.9 ± 9.9	0.371	28.6 ± 8.8	32.6 ± 10.7	31.8 ± 9.4	0.168
MUFA (g/day)	27.9 ± 10.6	28.2 ± 10.9	0.624	26.4 ± 10.8	28.6 ± 9.8	27.3 ± 10.1	0.423
PUFA (g/day)	22.4 ± 10.5	22.6 ± 10.6	0.843	21.4 ± 8.6	21.8 ± 10.4	22.3 ± 11.3	0.235
Total fiber (g/day)	22.8 ± 2.7	24.1 ± 2.6	0.004	19.8 ± 1.5	23.7 ± 2.2	26.4 ± 2.9	< 0.001
Vitamin A (RAE/day)	770 ± 342	779 ± 348	0.007	712 ± 297	784 ± 347	821 ± 385	0.001
Vitamin C (mg/day)	132 ± 56	144 ± 62	0.001	119 ± 45	138 ± 53	154 ± 69	0.018
Vitamin E (mg/day)	14.0 ± 5.7	15.6 ± 6.0	0.169	13.7 ± 4.8	16.1 ± 6.7	15.4 ± 5.8	0.274
Folate (mcg/day)	269 ± 79	298 ± 82	0.021	228 ± 84	287 ± 74	297 ± 83	0.032
Calcium (mg/day)	948 ± 215	943 ± 220	0.725	954 ± 186	893 ± 218	987 ± 247	0.342
Zinc (mg/day)	11.9 ± 4.6	10.4 ± 5.0	0.094	9.4 ± 3.8	12.2 ± 4.7	11.3 ± 5.1	0.167
**Food groups**							
Meats (g/day)	91.6 ± 49.8	88.2 ± 43.5	0.101	79.6 ± 41.8	98.3 ± 53.4	87.5 ± 40.6	0.182
Grains (g/day)	363 ± 116	365 ± 119	0.117	325 ± 87	354 ± 146	412 ± 113	0.002
Fruits (g/day)	223 ± 91	240 ± 95	< 0.001	194 ± 85	241 ± 102	281 ± 94	< 0.001
Vegetables (g/day)	258 ± 80	265 ± 85	< 0.001	204 ± 71	276 ± 90	304 ± 83	< 0.001
Nuts (g/day)	5.7 ± 1.9	4.9 ± 2.0	0.214	4.8 ± 1.4	5.3 ± 2.1	4.5 ± 1.8	0.368
Legumes (g/day)	14.8 ± 5.7	15.0 ± 5.6	0.209	13.6 ± 5.8	14.9 ± 4.8	16.2 ± 6.4	0.007
Dairy products (g/day)	185 ± 56	186 ± 57	0.325	182 ± 45	190 ± 67	184 ± 54	0.143
Olive sources (g/day)	1.37 ± 0.58	2.09 ± 0.61	< 0.001	1.18 ± 0.32	1.89 ± 0.65	2.18 ± 0.87	< 0.001

Data are expressed as mean ± standard deviation.

Min and Max of DPI in the participants were 12.2 and 62.1, respectively.

*DPI* dietary phytochemical index, *RAE* retinol activity equivalents, *SFA* saturated fatty acids, *MUFA* monounsaturated fatty acid, *PUFA* polyunsaturated fatty acid.

^†^Tertile cut-points of DPI are as follows: first tertile, < 26.80; second tertile, 26.80 to 33.15; third tertile, > 33.15.

^‡^Obtained from independent samples *t*-test.

^§^Obtained from ANCOVA.

^#^Energy intake is adjusted for sex and age; all other values are adjusted for age, sex and total energy intake.

Being in the highest tertile of DPI was associated with significantly higher energy intake (*P* < 0.001). Those in the lowest DPI tertile had lower intakes of carbohydrates (*P* = 0.001), fiber (*P* < 0.001), vitamin A (*P* = 0.001), Vitamin C (*P* = 0.018), and folate (*P* = 0.032), compared with those in the highest tertile. Moreover, the individuals in the top tertile of DPI consumed more grains (*P* = 0.002), fruits (*P* < 0.001), vegetables (*P* < 0.001), legumes (*P* = 0.007), and olive sources (*P* < 0.001) compared to those in the lower tertile (Table 2). There were no significant differences in protein, total fat, saturated fatty acids (SFA), monounsaturated fatty acids (MUFA), polyunsaturated fatty acids (PUFA), vitamin E, calcium, zinc, meats, nuts, and dairy products intakes across DPI tertile.

### Association of DPI with KOA

Crude and multivariable adjusted odds ratios (95% CIs) for KOA across the tertiles of DPI are shown in Table 3. In the crude model, individuals in the highest tertile of DPI had 65% lower odds of having KOA compared to those in the lowest tertile (OR 0.35, 95% CI 0.19 to 0.67, *P*-trend = 0.001). These associations remained significant after adjustment for potential confounders including age, sex, physical activity, smoking, and supplement use (OR 0.37, 95% CI 0.19 to 0.73, *P*-trend = 0.004) (Model 1). After further adjustment for BMI, this inverse association between DPI and odds of KOA remained significant (OR 0.35, 95% CI 0.18 to 0.69, *P*-trend = 0.003) (Model 2).

**Table 3:** Crude and multivariable-adjusted odds ratios (95%CIs) for knee osteoarthritis across the tertiles of dietary phytochemical index (DPI).

Variables	Tertiles of DPI			*P*-trend^†^
	Tertile 1	Tertile 2	Tertile 3	
**Total**				
n (cases/controls)	53/30	40/43	31/51	
Cut points of DPI	< 26.80	26.80 to 33.15	> 33.15	
Crude	1.0	0.53 (0.28, 0.98)	0.35 (0.19, 0.67)	0.001
Model 1^‡^	1.0	0.56 (0.29, 1.09)	0.37 (0.19, 0.73)	0.004
Model 2^§^	1.0	0.54 (0.28, 1.05)	0.35 (0.18, 0.69)	0.003
**Males**				
n (cases/controls)	27/12	17/22	14/21	
Cut points of DPI	< 26.72	26.72 to 32.82	> 32.82	
Crude	1.0	0.37 (0.15, 0.95)	0.31 (0.12, 0.81)	0.016
Model 1	1.0	0.36 (0.13, 0.97)	0.30 (0.11, 0.84)	0.021
Model 2	1.0	0.29 (0.10, 0.85)	0.24 (0.08, 0.71)	0.009
**Females**				
n (cases/controls)	26/18	23/21	17/30	
Cut points of DPI	< 26.86	26.86 to 33.44	> 33.44	
Crude	1.0	0.64 (0.28, 1.47)	0.39 (0.17, 0.84)	0.029
Model 1	1.0	0.78 (0.31, 1.87)	0.44 (0.18, 0.92)	0.046
Model 2	1.0	0.79 (0.31, 1.79)	0.42 (0.15, 0.90)	0.040

*DPI* dietary phytochemical index.

^†^Obtained from the Mantel–Hanszel extension test.

^‡^Adjusted for age, sex, physical activity, smoking and supplement use.

^§^Further adjuster for BMI.

Although, the interaction of DPI and sex was not significant, still, we carried out a stratified analysis by sex (Table 3). When stratified by sex, the DPI score was inversely associated with the chance of KOA in both males and females. In the crude model, males (OR 0.31, 95% CI 0.12 to 0.81, *P*-trend = 0.016) and females (OR 0.39, 95% CI 0.17 to 0.84, *P*-trend = 0.029) in the highest tertile of DPI had 69% and 61% lower odds of having KOA compared to those in the lowest tertile, respectively. After adjustment for potential confounders (Model 1), this inverse relationship remained significant in both sexes. Further adjustment for BMI (Model 2) did not change this inverse association between DPI and the odds of KOA neither in males nor in females.

## Discussion

The results of the present case–control study showed that the highest tertile of DPI was associated with lower odds of KOA in both genders, but this association was stronger among males than females. To the best of our knowledge, this is the first case–control study that evaluated the association between DPI and the odds of KOA.

KOA is a multifactorial disease that is impacted by both mechanical and biological factors^35^. In the present study, we indicated that men in the highest tertile of DPI were 76% less likely to have KOA compared to those in the lowest tertile. Also, compared to those in the lowest tertile of DPI, women in the highest tertile of DPI were 58% less likely to have KOA. All study participants were found to have 65% lower odds of KOA when compared to those in the lowest tertile of DPI.

DPI has been linked to some chronic and autoimmune diseases, including cardiovascular disease^36^, diabetes^37^, psychological distress^38^, Alzheimer’s disease^39^, and inflammatory bowel disease^40^. However, there is no evidence of the association between DPI and the odds of KOA. A meta-analysis which was conducted in 2021 on 97,157 individuals, indicated that vegetarian diets, which are rich in phytochemicals, were associated with a lower odds of KOA (OR 0.71 [95% CI 0.45, 0.97])^41^. Moreover, healthy dietary patterns rich in phytochemicals, similar to what DPI represents, were found to be associated with lower odds of KOA. Veronese et al.^42^ reported that participants with higher adherence to the Mediterranean diet had a significantly lower prevalence of KOA compared to those with lower adherence (Q4: 25.2% vs. Q1: 33.8%; *P* < 0.0001).

There are some possible mechanisms available that higher intake of phytochemicals is associated with lower OA progression although the exact mechanism is unclear^43^. According to researchers, OA progresses because of an imbalance between cartilage matrix anabolic repair and catabolic breakdown^44^. Based on the evidence, dietary polyphenols, as the biggest group of phytochemicals, may have favorable effects in the management of inflammatory arthritis and may therefore benefit people with OA^43^. The anti-osteoarthritic potential of dietary polyphenols seems to be mediated through down-regulating the inflammatory cytokines, as well as reducing oxidative stress because of their anti-inflammatory and ant-oxidative properties^45^. Based on the literature, elevated levels of inflammatory cytokines in OA, contribute to the production of matrix metalloproteinases (MMPs) by cartilage chondrocytes which leads to the cartilage breakdown^46^. Several studies have shown that polyphenol-rich foods may reduce serum levels of IL-1, IL-6, MMP-1, and MMP-13 in patients with KOA^47,48^. Some evidence proposes that polyphenols appear to have inhibitory effects on inflammatory responses and the related signaling pathways which may contribute to the reduced production of COX-2 and MMPs to slow the catabolic breakdown of the cartilage matrix^45^. Furthermore, it has been found that 12 weeks’ consumption of soy isoflavones, as polyphenolic compounds, resulted in increased serum IGF-1 levels^49^, which appeared to have beneficial effects on cartilage matrix formation via growth factors^50^. The increased prevalence of OA after menopause suggests an association between estrogen levels and OA development^51^. Because of the similar structure of isoflavones and estrogen, isoflavones intake may have a favorable effects on OA^52^.

This study had several strengths. To the best of our knowledge, this is the first study examining the association between DPI and odds of KOA. In the current analysis, we adjusted for several potential confounders. A validated FFQ was used to determine data on the usual dietary intakes of participants. A holistic approach has also been used in this study, rather than focusing on a single nutrient. However, our study had some limitations that should be addressed. First, including the relatively young participants in the present study may preclude generalizing the findings to people outside of this age range (20–60 years). Second, measurement errors might lead to erroneous categorizations regarding phytochemical consumption by individuals. Third, DPI may contain different elements in different regions, which makes our findings inapplicable in all regions. Forth, in the present study other risk factors related to KOA such as history of knee injury, physically demanding job, bone deformity, etc. had not been examined. In addition, due to the case–control design of the study, a cause-and-effect relationship cannot be obtained from the results. So, further research including prospective or longitudinal studies are needed to confirm these findings.

## Conclusion

The present case–control study indicated that DPI and KOA have inverse associations. Consequently, increasing the consumption of foods with high phytochemical content including fruit, vegetables (i.e. broccoli, cabbage, collard greens, kale, cauliflower, and Brussels sprouts), and whole grains may help reduce the odds of KOA.

## Data Availability

The data that support the findings of this study are available from the corresponding author upon reasonable request.
